# Therapeutic Role of Additional Mirror Therapy on the Recovery of Upper Extremity Motor Function after Stroke: A Single-Blind, Randomized Controlled Trial

**DOI:** 10.1155/2022/8966920

**Published:** 2022-12-31

**Authors:** Xin Wen, Li Li, Xuelian Li, Huanghong Zha, Zicai Liu, Yang Peng, Xuejin Liu, Huiyu Liu, Quan Yang, Jing Wang

**Affiliations:** ^1^Department of Rehabilitation Medicine, Yue Bei People's Hospital, Shaoguan, Guangdong, China; ^2^School of Rehabilitation Medicine Gannan Medical University, Ganzhou, Jiangxi, China; ^3^Yue Bei People's Hospital, Shaoguan, Guangdong, China; ^4^Department of Neurology Medicine, Yue Bei People's Hospital, Shaoguan, Guangdong, China

## Abstract

**Background:**

Rehabilitation of upper extremity hemiplegia after stroke remains a great clinical challenge, with only 20% of patients achieving a basic return to normal hand function. How to promote the recovery of motor function at an early stage is crucial to the life of the patient.

**Objectives:**

To invest the effects of additional mirror therapy in improving upper limb motor function and activities of daily living in acute and subacute stroke patients, and further explore the effects of other factors on the efficacy of MT.

**Methods:**

Participants who presented with unilateral upper extremity paralysis due to a first ischemic or hemorrhagic stroke were included in the study. They were randomly allocated to the experimental or control group. Patients in the control group received occupational therapy for 30 minutes each session, six times a week, for three weeks, while patients in the experimental group received 30 minutes of additional mirror therapy based on occupational therapy. The primary outcome measures were Fugl-Meyer Assessment—upper extremity (FMA-UE), Action Research Arm Test (ARAT), and Instrumental Activity of Daily Living (IADL) which were evaluated by two independent occupational therapists before treatment and after 3-week treatment. A paired *t*-test was used to compare the values between pretreatment and posttreatment within an individual group. Two-sample *t*-test was utilized to compare the changes (baseline to postintervention) between the two groups.

**Results:**

A total of 52 stroke patients with unilateral upper extremity motor dysfunction who were able to actively cooperate with the training were included in this study. At baseline, no significant differences were found between groups regarding demographic and clinical characteristics (*P* > 0.05 for all). Upper limb motor function and ability to perform activities of daily living of the patients were statistically improved in both groups towards the third week (*P* < 0.05). In addition, statistical analyses showed more significant improvements in the score changes of FMA-UE and IADL in the experimental group compared to the control group after treatment (*P* < 0.05), but no significant difference was observed in the ARAT score changes between the two groups (*P* > 0.05). The subgroup analysis showed that no significant heterogeneity was observed in age, stroke type, lesion side, and clinical stage (*P* > 0.05).

**Conclusion:**

In conclusion, some positive changes in aspects of upper limb motor function and the ability to perform instrumental activities of daily living compared with routine occupational therapy were observed in additional mirror therapy. Therefore, the application of additional mirror therapy training should be reconsidered to improve upper extremity motor in stroke patients.

## 1. Introduction

Almost 80% of acute stroke patients have upper extremity motor dysfunction [1]. Upper extremity paralysis is likely to largely improve within 6 months [2], however, up to 50-60% of stroke survivors remain persistent with upper extremity motor dysfunction at 6 months [3]. Persistent upper extremity dysfunction affects many poststroke patients and is strongly associated with decreased activities of daily living and poor quality of life [4, 5]. Therefore, early exercise interventions are necessary to improve the patients' upper limb motor function and improve their activity of daily living.

Despite some trials have reported that treatments such as robot-assisted training [6] and constraint-induced movement therapy [7], functional electrical stimulation [8], and repetitive transcranial magnetic stimulation [9] can facilitate upper limb motor function recovery, some of these interventions require expensive equipment or active interaction with a therapist and difficult to apply to home rehabilitation on a large scale. Moreover, most of the rehabilitation measures are limited to the hospital, and many of them are not available when the patient is discharged. In many cases, the motor function regained in treatment deteriorates over time when the rehabilitation therapy is discontinued [10]. Thus, consistent and effective treatment that can be easily performed at home is essential for the patient's functional recovery.

Mirror therapy (MT) is suggested as an accessible and low-cost alternative intervention to improve the motor function of patients [11, 12]. Altschuler et al. [13] proposed MT for the rehabilitation of poststroke hemiplegia, and they observed an improvement in motor performance in patients with chronic stroke, and a new chapter of research on MT for the treatment of upper extremity movement disorders after stroke was opened. Several mechanisms for the effects of MT on motor function recovery after stroke have been proposed. A mirror was used to present the subject with a mirror reflection of the normal movements of the unaffected arm as if it was affected. The continuous mirror visual feedback stimulated the main motor cortex of the brain, thus affecting the electrical activity and excitability of the cortex [14, 15, 16], promoting the remodeling of brain function and facilitating the recovery of motor function. The therapeutic effect of MT is also attributed to the activation of the mirror neurons system (MNS). The MNS is located mainly in the occipital, temporal, and parietal visual-related areas and in the frontoparietal motor areas on both sides, connecting sensory neurons for visual processing and motor neurons for action signaling [17]. Nojima et al. [14] confirmed by transcranial magnetic stimulation that the improvement of motor function after MT training was more associated with the remodeling of the major motor cortical areas. In MT training, constant visual and somatosensory stimulation could activate the MNS, induce neural remodeling [18, 19], and cause upper motor function recovery. In addition, in MT, patients performed bilateral upper limb motor training independently or with assistance, and motor cortical areas were extensively activated when bilateral limbs performed symmetrical movements [20, 21], and it can be assumed that mirror visual feedback could ease some of the motor pathways on the affected side and promote the recovery of limb motor function.

Several previous studies [22, 23, 24, 25] have shown beneficial effects in improving the upper limb motor function in the acute to chronic phase of stroke. Gurbuz et al. [26] investigated the effects of MT combined with routine rehabilitation training on upper limb motor and functional recovery in patients after stroke, and they found that MT provided additional benefits in motor recovery of the upper limb in patients. However, the sample size of this study was small, with only 31 patients. A study by Lim et al. [27] indicated that MT containing functional tasks played a significant role in improving the upper limb functions and the ability to perform activities of daily living among patients with subacute stroke. A meta-analysis has suggested that MT could improve the upper limb motor function in subacute and chronic stroke patients, but with no significant difference in the improvements between subacute and chronic patients [28]. However, this meta-analysis only provided an indirect comparison of the effects of treatment in subacute and chronic stroke patients, and its accuracy needs to be further verified. It has been suggested that MT training has a variable effect on ADL in patients with left- and right-sided hemiplegia, with a significant increase in motor function in subacute stroke patients, with right-sided upper limb paralysis and no significant improvement in patients with left-sided upper limb paralysis [29].

Based on the above background, the purpose of this observer-blind, randomized controlled trial was to assess the beneficial impacts of MT on the recovery of upper limb motor function in acute and subacute stroke patients and further conduct subgroup analysis to explore the effects of other factors on the efficacy of MT to address gaps in previous studies.

## 2. Methods

### 2.1. Participants

Participants were identified and selected from acute and subacute stroke patients hospitalized in the Department of Rehabilitation Medicine, Yuebei People's Hospital between August 2021 and May 2022. The inclusion criteria were the following: (1) first-ever unilateral ischemic or hemorrhage stroke, confirmed by computed tomography or magnetic resonance imaging; (2) date of stroke less than 6 months; (3) moderate to severe upper extremity motor dysfunction (FMA-UE [30] scores <40); (4) aged between 18 and 80 years with no serious cognitive impairment; (5) no severe vision or visuospatial neglect [31] (the best gaze and visual subtest in National Institutes of Health Stroke Scale (NHISS) = 0 [32]); (6) right-handed; and (7) no depression (Hamilton Depression Rating Scale (HAMD) [33] scores <8) with good compliance. Exclusion criteria were (1) have other neurologic, neuromuscular, or orthopedic disease affecting upper motor function; (2) excessive spasticity of the elbow flexion muscles (Modified Ashworth Scale [34] > 3); (3) recurrence of stroke or epilepsy during the study period; (4) serious systemic impairment or concomitant diseases; and (5) patients refused to participate in the experiment. All participants were required to sign an informed consent form before participation in the study. The experiment was reviewed and approved by the Ethics Committee of the Yuebei People's Hospital (Approval number: KY-2021-097).

### 2.2. Sample Size Estimation

The sample size was estimated prospectively using PASS software version 22.0.2 (NCSS, LLC. Kaysville, Utah, USA). Based on a previous randomized controlled study [35], we expected an effect size of 0.49, with an *α* level of 0.05 and a power of 0.8; the minimum sample size was *n* = 40 (20 per group). To allow for dropouts, we planned to recruit an additional 10 participants. Thus, the planned sample size was 50 (25 subjects per group).

### 2.3. Study Design

An assessor-blind, randomized controlled study was designed. Participants who met the criteria were randomly assigned to the experimental or control group. The allocation ratio was 1 : 1. A computer-generated completely randomized digital table was used to generate the random allocation sequence. Two certified occupational therapists were responsible for the clinical assessments but were blinded to the group assignment throughout the study. All participants received functional assessments before starting the treatment and after 3-week of treatment. This study protocol was registered with the Chinese Clinical Trial Registry (Registration number: ChiCTR2200055439). The experiment was implemented and reported according to the Consolidated Standards of Reporting Trials guidance [36].

### 2.4. Intervention

All included patients received routine stroke rehabilitation treatment for 3 weeks, 6 days a week, and half an hour per day. The routine treatment included neurodevelopmental facilitation techniques, physiotherapy, occupational therapy, and speech and swallowing training (if necessary). Patients in the experimental group received an extra 30 minutes of MT per day based on the routine stroke rehabilitation treatment.

### 2.5. Mirror Therapy

**Figure 1:**
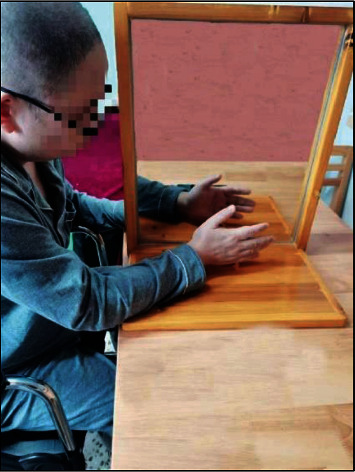
Mirror therapy.

In the first intervention, patients were introduced to the general procedures of MT. The MT procedures were as follows: patients were asked to sit in front of a table of appropriate height with their arms resting on the table and a mirror (35 cm × 35 cm) placed between the patient's arms. The nonaffected arm was placed in front of the mirror and the affected arm was placed and obscured behind the mirror (Figure 1). The patient was instructed by a therapist to complete the movement of the nonaffected arm while staring at the reflection of the uninvolved arm in the mirror as if it was the involved one. Simultaneously, subjects were required to conduct the same movements by the involved arm as actively as possible, if necessary; the therapist assisted the patients with the movements of the affected arm. The movements consisted of forearm rotation, elbow, wrist, and finger flexion and extension movements, and hand grasping. Selected appropriate movement tasks according to the function of the affected upper limb. All patients in the experimental group participated in MT for 30 minutes per day, six days per week, for 3 weeks.

### 2.6. Clinical Assessment

National Institute of Health Stroke Scale (NHISS) and Hamilton Depression Rating Scale (HAMD) were used to assess at baseline. NIHSS was used to assess the degree of functional impairment caused by stroke, and HMAD was evaluated to assess depression in patients. Measurements of the Fugl-Meyer Assessment—Upper Extremity (FMA-UE), Action Research Arm Test (ARAT), and Instrumental Activity of Daily Living (IADL) were evaluated at baseline and postintervention (after 3 weeks). The primary motor function measure was FMA-UE, and the secondary measures were ARA and IADL. These clinical assessments were recorded by two dependent occupational therapists.

The primary motor measure was the score changes of FMA-UE from baseline to postintervention. The Fugl-Meyer scale was developed to evaluate recovery after stroke in the shoulder, elbow, forearm, wrist, and hand [37, 38]. The FMA-UE included 33-item upper limb activities; each rated from 0 to 2, with 0 representing “cannot conduct,” 1 representing “conducts partially,” and 2 representing “conducts completely”. The total score of the FMA-UE subscale varies from 0 to 66, with higher scores presenting better function. The reliability for FMA-UE was high (overall intraclass correlation coefficient = 0.96), and the intraclass correlation coefficient for the upper extremity subsection was 0.97 [39].

The second motor outcome was ARAT [40]. ARAT was developed to assess the activity and participation of the upper limb in stroke patients. ARAT has high validity and reliability [40, 41]. The ARAT included 19 items subdivided into four subsets: grasp, grip, pinch, and gross motor [42]. Each item was scored from 0 (indicating no movements) to 3 (indicating performs completely). The maximum score of the ARAT was 57.

The other secondary outcome was IADL. IADL was utilized to evaluate the level of functional independence. This questionnaire consisted of 8 items: the capability of using the telephone, going shopping, preparing food, doing housework, washing personal clothes, using transportation, being responsible for taking medications, and addressing finance [43, 44, 45]. The maximum score of the IADL was 24. The higher scores represented greater independence.

### 2.7. Statistical Analyses

In this present trial, statistical analyses were conducted in SPSS software (SPSS 28.0, Chicago, IL, USA)), and suitable descriptive statistics were performed to summarize the general characteristics at baseline of the patients. The measurement data obtained in this study were presented as mean ± SD (standard deviation) or median (interquartile range). The normality of the variables was assessed by using the Shapiro-Wilk test. Chi-square or two-sample *t*-test or Mann–Whitney test was applied to check for any significant group differences in demographics and outcome variables between the two intervention groups before starting the intervention. A paired *t*-test was used to compare the values between pretreatment and posttreatment within an individual group. Two-sample *t*-test was utilized to compare the changes (baseline to postintervention) between the two groups. Every outcome was regarded as an independent domain, and we only observed the effect of MT on each domain independently. Subgroup analyses were conducted by dividing the sample into two groups based on their age (less or more than 60 years old), their stroke type (ischemic or hemorrhagic), their lesion side (right or left hemisphere), and clinical stage (less than 1month or more than 1 month), respectively. A post hoc age, stroke type, lesion side, and clinical stage subgroup analysis was conducted by two independent *t*-test to investigate the effects of other factors on the efficacy of MT.

## 3. Results

**Figure 2:**
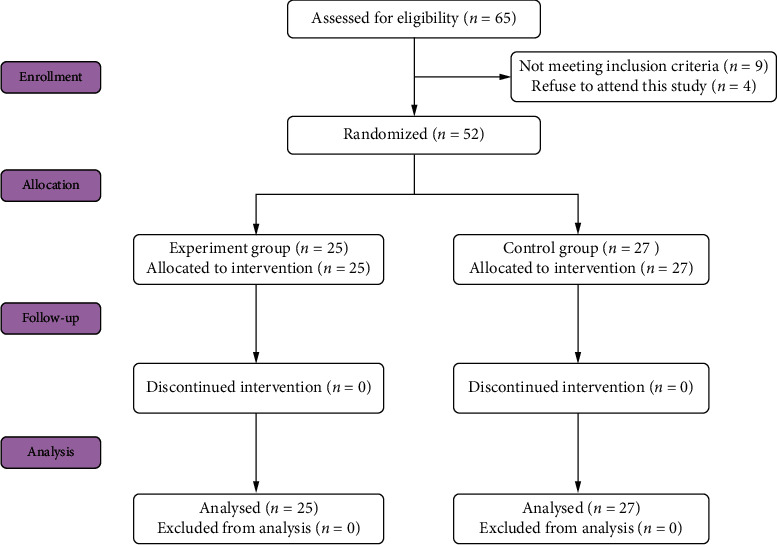
Flow diagram of subjects through the study.

Figure 2 shows the flow chart of the present trial and the exact number of participants. During the recruitment period, a total of sixty-five patients were screened from August 2021 to May 2022, 9 patients did not meet the study eligibility criteria and 4 patients refused to attend this study. In total, 52 participants were enrolled in our trial. All 52 patients completed the assessment and a 3-week treatment without any side effects, and no adverse events happened during the treatment period. 25 patients (aged 53.76 ± 11.76 years, 18 males and 7 females) received additional MT training based on occupational therapy (experimental group) and 27 patients (aged 57.89 ± 10.74 years, 23 males and 4 females) received occupational therapy (control group). None of the patients discontinued the experiment during the intervention.

The overall characteristics of the subjects were summarized in Table 1. There were no significant differences in the distribution of age, gender, lesion type, lesion side, duration after stroke onset, hypertension, diabetes, hyperlipidemia, and the scores of NHISS, and HMAD at baseline between the two groups (*P* > 0.05) (see Table 1). For the upper limb motor function assessment at baseline, no significant differences in the scores of FMA-UE, ARAT, and IADL between the experimental and control groups were found (*P* > 0.05) (see Table 1).

**Figure 3:**
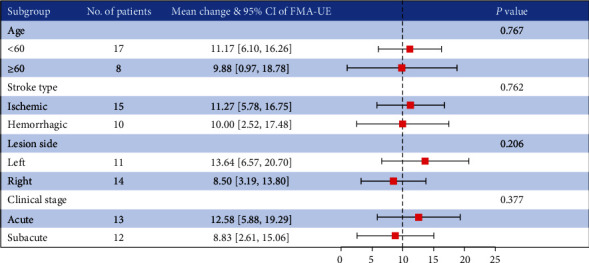
The subgroup analysis of age, stroke type, lesion side, and clinical stage. Clinical stage: Acute: < 1 month; Subacute: 1 to 3 months.

After the intervention, the experimental group presented significant improvements in the scores of FMA-UE, ARAT, and IADL (*P* ≤ 0.001 for all) (see Table 2). The control group also presented significant improvements in the scores of FMA-UE, ARAT, and IADL (*P* < 0.05 for all) (see Table 2). In addition, statistical analyses showed more significant improvements in the score changes of FMA-UE and IADL in the experimental group compared to the control group after 3-week of treatment (*P* < 0.01) (see Table 3). However, there was no significant difference in the score changes of ARAT between the two groups (*P* > 0.05) (see Table 3). The subgroup analysis showed that there was no significant difference in age, stroke type, lesion side, and clinical stage (*P* = 0.767, 0.762, 0.206, and 0.377, respectively) (see Figure 3).

## 4. Discussion

This randomized controlled trial explored whether MT combined with conventional therapy affects upper extremity motor function and activities of daily living in stroke patients with upper extremity motor dysfunction in comparison to conventional therapy. In addition, we also conducted subgroup analyses to attempt to investigate the effects of other factors on the efficacy of MT. Both experimental and control groups had improvements in motor function, participation, and activities of daily living as FMA-UE, ARAT, and IADL scales. Noting that, the patients in the experimental group showed more significant improvements in the score changes of FMA-UE and IADL than the control group after treatment. However, there was no significant difference in the improvement in ARAT between patients in the experimental and control groups.

From the results of statistical analyses, we found that both MT and conventional occupational therapy were beneficial for the improvement of the upper extremity motor function after stroke. Consistent with previous studies [46, 47], our study observed more significant motor function improvement in acute and subacute stroke patients after additional MT training than after conventional occupational therapy. Michielsen et al. [11] found that MT has some effects on chronic stroke patients. A study by Colomer et al. [25] also declared that MT provided limited but positive effects on light touch sensitivity in chronic stroke survivors with severely impaired upper-limb function. Furthermore, we also found that after MT training, there was a significant improvement in activities of daily living compared to traditional occupational therapy. This result is in accordance with these previous studies [12, 48, 49] and in inconsistent with the study of Wu et al. [50]. The reason why our findings differed from Wu et al. may be due to the different duration after stroke onset in the included patients. In our study, we selected patients within 6 months after stroke onset, while Wu et al. selected stroke patients with onset of more than 6 months. This may suggest that MT could significantly improve the activities of daily living of patients within 6 months, while it is less effective in patients more than 6 months after stroke onset. In our study, we demonstrated that the application of MT training should be reconsidered to enhance motor performance and improve the activities of daily living in stroke patients with upper extremity motor dysfunction. The recovery of motor function depends largely on neuroplasticity changes [51], and conventional therapy can promote functional reorganization of the brain, which is an effective way to reduce the disability rate [52]. However, patients are inattentive and have low initiative when receiving conventional treatment, which is detrimental to the activation of the corresponding cerebral cortex, which in turn affects neuroplasticity and functional reorganization [53]. MT requires active movement of bilateral upper limbs, which can increase the patient's initiative, and requires simultaneous observation of the mirror reflection of normal limb movement, which can make the patient's attention more focused, and will be more conducive to cortical activation than conventional rehabilitation training. Therefore, both MT and conventional therapy can promote the recovery of motor function to some extent, and this combination of MT and conventional therapy can compensate for the shortcomings of conventional therapy and further improve the patient's motor function.

It is well known that brain excitability with aging, which may affect the recovery of motor function. According to the World Health Organization (WHO), people over 60 years of age are defined as elderly, thus we chose 60 years as the cutoff and divided the patients into two groups. No significant difference in the recovery of motor function between the two groups, and the results of this study were similar to those of Falconer et al. [54]. They indicated that the older (> 75 years) group's motor index scored significantly lower than both the young (< 65 years) and young-old (65-74 years) groups, and no significant difference was observed between the young and young-old group. We also found patients with ischemic stroke seem to have better improvement in mean changes of FMA-UE scores than patients with hemorrhagic stroke. We observed that patients injured in the left hemisphere have better mean changes of FMA-UE scores than those in right, with a difference of 5.14 points. We believe that this result may be related to the dominant hemisphere of the brain. Patients in acute seemed to be better compared to patients in subacute. However, all of these differences were qualitative observations not supported by statistical analysis. Currently, it has been recognized that the best time for recovery of motor function was within 3 months after the stroke onset, and intervening as early as possible could promote functional recovery to a certain degree [55, 56].

In this present study, we chose patients with unilateral upper extremity paralysis within six months after stroke onset. It did not exclude the possibility of spontaneous recovery of the patient, because partly patients may recover spontaneously their motor function in weeks after stroke onset [57]. Both the experimental and control groups improved motor performance, in part attributed to the effects of spontaneous recovery and conventional occupational therapy [58]. However, the mean change values of scores in the experimental group were all significantly higher than those in the control group, with the experimental group being more effective compared to the control group. It suggested that MT could improve upper limb motor performance after stroke within six months. What is more, participants were not blinded, which may also have an impact on the treatment of MT. Patients in the experimental group underwent additional MT training while the control group did not receive placebo treatment, which may have exaggerated the effect of mirror treatment.

In our study, patients received 30 minutes of additional MT training per day, six times a week for a total of three weeks. At present, there is no complete consensus on the daily intervention time, frequency, modality, and specific efficacy of MT interventions. It has been shown that both daily interventions ≤30 minutes [27, 59] and daily interventions >30 minutes [26, 35] could improve upper extremity motor function. However, no clinical trials have explored the impacts of different daily treatment times on recovery of motor function. Only a meta-analysis [60] indicated that a daily intervention time of MT ≤ 30 minutes was more favorable for the functional recovery of patients.

However, our study has several limitations. Firstly, despite no statistical differences in demographic features and baseline assessments between the two groups, the experimental group was younger, with more hemorrhagic stroke, and had higher scores on ARAT than the control group. Differences in the study groups (although not significant) may lead to overestimating changes after MT training. Secondly, participants included in our study were not blinded, and the control group did not undergo placebo treatment, which did not exclude that this difference in treatment effect was due to the unequal duration of treatment. In a future study, the control group could be subjected to the same training procedure as the experimental group in the absence of a mirror. Besides, although the relatively large number of patients, this was a single-center study. We adopted clinical rating scales to assess the upper limb motor function based on clinical judgments, which may lead to a subjective evaluation. Furthermore, only patients with good compliance were included in our study, and the effect of MT on patients with poor adherence was unknown. In future studies, the potential effect of compliance as an important factor in the therapeutic effect of MT could be explored. Finally, we performed the 3-week treatment without follow-up, and the long-term and sustained therapeutic effects of MT were unclear. A large-scale, multiple-center randomized controlled study should be performed to explore the long-term and sustained effects of MT on stroke patients in the future.

## 5. Conclusion

In summary, MT combined with conventional occupational therapy can effectively improve upper extremity motor performance and enhance the ability to perform activities of daily living in stroke patients, and MT can be considered as an adjunctive treatment for upper limb motor function rehabilitation within 6 months of the onset of the stroke. However, MT did not seem to have an additional effect in improving the activity participation component of the stroke patients at least in the present study.

## Figures and Tables

**Table 1:** Baseline characteristics of patients included in the study.

Characteristic	Randomized		*P* value
	Experimental group (*N* = 25)	Control group (*N* = 27)	
Age (years)	53.76 ± 11.76	57.89 ± 10.74	0.192^a^
Gender (male/female)	18/7	23/4	0.317^b^
Lesion type (ischemic/hemorrhagic)	15/10	21/6	0.232^b^
Lesion side (left/right)	11/14	12/15	1.000^b^
Duration after stroke onset (days)	31.00 (16.50-50.50)	30.00 (21.00-60.00)	0.533^c^
Hypertension (yes/no)	17/8	20/7	0.762^b^
Diabetes (yes/no)	4/21	8/19	0.329^b^
Hyperlipidemia (yes/no)	10/15	6/21	0.232^b^
NHISS	8.28 ± 3.99	7.22 ± 3.27	0.299^a^
HAMD	2.64 ± 2.38	3.29 ± 2.69	0.339^c^
FMA-UE	19.48 ± 16.62	20.59 ± 18.65	0.706^c^
ARAT	6.16 ± 12.43	4.44 ± 9.29	0.905^c^
IADL	9.16 ± 3.57	9.81 ± 3.22	0.491^a^

Data were presented as mean ± SD (standard deviation) or median (interquartile range). Abbreviations: NHISS: National Institute of Health Stroke Scale; HAMD: Hamilton Depression Rating Scale; FMA-UE: Fugl-Meyer Assessment Upper Extremity subscale; ARAT: Action Research Arm Test; IADL: Instrumental Activity of Daily Living. *P*^a^, two independent-sample *t-*test was used to compare two groups in normal distribution variables; *P*^b^, chi-squared test; *P*^c^, Mann–Whitney test was used to compare two groups in nonnormal distribution variables.

**Table 2:** The scores of FMA-UE, ARAT, and IADL at baseline and posttreatment in two groups.

Outcomes	Experimental group		*t* value	*P* value	Control group		*t* value	*P* value
	Baseline	Posttreatment			Baseline	Post-treatment		
FMA-UE	19.48 ± 16.62	30.24 ± 18.67	4.11	< 0.001	20.59 ± 18.65	25.04 ± 18.98	5.99	< 0.001
ARAT	6.16 ± 12.43	10.12 ± 15.70	3.25	0.001	4.44 ± 9.29	6.11 ± 9.35	3.26	0.003
IADL	9.16 ± 3.57	12.40 ± 3.94	7.11	< 0.001	9.81 ± 3.22	11.00 ± 3.26	4.05	< 0.001

Data were expressed as mean ± SD (standard deviation). Abbreviations: FMA-UE: Fugl-Meyer Assessment Upper Extremity subscale; WMFT: Wolf Motor Function Test; ARAT: Action Research Arm Test; IADL: Instrumental Activity of Daily Living. A paired *t-test* was utilized to compare the values between baseline and post-treatment within two groups.

**Table 3:** The comparison of delta scores of measures between two groups after intervention.

Outcomes	Experimental group	Control group	*t* value	*P* value
*Δ* FMA-UE	10.76 ± 9.93	4.44 ± 3.86	2.979	0.006
*Δ* ARAT	3.96 ± 7.07	1.70 ± 2.64	1.502	0.144
*Δ* IADL	3.24 ± 2.28	1.19 ± 1.52	3.853	< 0.001

Data are mean ± standard deviation. Abbreviations: FMA-UE: Fugl-Meyer Assessment Upper Extremity subscale; ARAT: Action Research Arm Test; IADL: Instrumental Activity of Daily Living. Two-sample *t* test was performed to compare the differences in score changes between the experimental and control groups after intervention.

## Data Availability

Ask corresponding author for original data.
